# Enhancing electrical conductivity of coal-derived graphite by boric acid catalysis: Correlating 2H/3R phase ratio with crystallite structure

**DOI:** 10.1371/journal.pone.0347483

**Published:** 2026-04-24

**Authors:** Xiaomei Zhang, Xinyu Chen, Yeersheng Jiangbaolati, Shanshan Liu, Jidun Sha, Xiaoming Zhang, Hao Chen, Shuo Feng

**Affiliations:** 1 School of Geology and Mining Engineering, Xinjiang University, Urumqi, Xinjiang, China; 2 Xinjiang Key Laboratory of Continental Dynamics and Ore Prediction in the Xinjiang-Central Asia Orogenic Belt, Urumqi, Xinjiang, China; 3 Mineral Experimental Research Center, Xinjiang Bureau of Geology (Xinjiang Quality Testing Centre of Gemstones), Urumqi, Xinjiang, China; 4 School of Natural Resources Science and Technology, Xinjiang University of Technology, Hetian, Xinjiang, China; National Chung Cheng University, Taiwan & Australian Center for Sustainable Development Research and Innovation (ACSDRI), AUSTRALIA

## Abstract

Natural graphite scarcity and conventional catalyst-induced defects limit the scalable production of high-performance coal-derived graphite. Herein, we demonstrate boric acid (H_3_BO_3_) as a green catalyst for coal graphitization by comparing with Fe_2_(SO_4_)_3_, FeCl_3_, FeS_2_, and H_3_BO_3_+FeCl_3_, with a focus on crystallite structure, 2H/3R polytypic graphite, and electrical conductivity. X-ray diffraction (XRD) analysis reveals that H_3_BO_3_ catalysis significantly elevates structural order, presenting a narrow and sharp (002) band, and increasing in-plane crystallite size (La), stacking height (Lc), and the proportion of graphite (f_ɑ_) in the bulk sample. High resolution transmission electron microscopy (HR TEM) identifies graphite-like nanostructures and planar graphene nanostructure as the key contributor to electrical conductivity. A linear relationship between La and 2H/3R is established, associated with electrical conductivity, confirming the intrinsic correlation between graphite-like nanostructures and crystalline size. This work clarifies the feasibility of boric acid, providing a low-cost and efficient strategy for fabricating high-performance coal-derived graphite, which holds great potential for applications in energy storage and conductive fields.

## 1 Introduction

Graphite and graphite-derived carbon materials have attracted great interest in fields of energy storage, conductive electrode, and lithium-ion batteries etc., driven by global demand for low-carbon and high-efficiency [1–4]. So, graphite is listed as strategic resource by numerous countries and zones [1,3]. Natural graphite is expensive in purification and unstable in supply. Coal is a cost-effective and sustainable feedstock for preparing carbon materials, such as graphite, graphene, carbon dots, and porous carbons [1,5,6]. And coal is abundant in reserves, rich in carbon content, and composed of aromatic structural units, benefiting for graphitization. However, the intrinsic disorder of coal limits its graphitization [6–11], which severely limits the practical application of coal-based carbonaceous materials.

Catalytic graphitization is widely recognized as pivotal and indispensable method to overcome the inherent drawbacks of coal when utilized as a carbon feedstock. Catalyst can reduce the activation energy for the structural alignment and rearrangement [1,2,12–14], thereby facilitating the structure order of crystalline structure. The crystalline structure governs the intrinsic properties of coal and the final performance of products. Metal catalysts employed in coal graphitization, predominantly including Fe_2_O_3_, Fe_2_(SO_4_)_3_, FeCl_3_, FeS_2_, and other metal oxides, have been extensively investigated [1,2,14]. Kim K. J. et al. prepares a highly crystalline graphite from sub-bituminous coal using an Fe_2_O_3_ as a catalyst [1], which provides great potential for coal utilization. Nevertheless, these catalysts still can introduce metal impurities and need to conduct further procedures for recovery [1]. In contrast, boric acid (H_3_BO_3_) has attracted attention as a potential catalyst for coal graphitization, owing to its ability to modulate aromatic layer orientation and reduce interlayer defects without introducing metal impurities [2,14–17]. Boric acid can help producing aromatic hydrocarbons and cause thermal condensations, thus improving the crystallinity of produced graphite [2]. For example, catalytic graphitization of boric acid added pitch [2] and polyacrylonitrile carbon fibers [14] have been investigated and the products show great electrochemical and mechanical performances. However, the catalytic graphitization of coal is different from those of pitch coke, petroleum coke due to different crystalline structure and structural heterogeneity of coal [1,18]. Furthermore, the rhombohedral (3R) and hexagonal (2H) phases of graphite exhibit different electronic properties [4,19,20]. The relationships between crystallite structure and product performance of coal-based graphite still require further exploration [14,19], which is essential for preparing high quality graphite from coal.

This work investigates the crystallite structure, nanostructure, and electrical conductivity of coal-based graphite with boric acid as a catalyst. This work aims to demonstrate the potential of boric acid in catalyzing coal graphitization and to clarify the relationship between crystalline structure and material properties. The findings offer an economical and environmental way to produce high-quality coal-based graphite in energy storage and conductive applications.

## 2 Materials and methods

### 2.1 Materials and pretreatment

Three coals (named C1-25, C2-25, and C3-25), a sub-bituminous coal and two anthracites, were formed from the Jurassic, Permian, and Carboniferous systems, from western China. The coal was pulverized to below 200 meshes. Minerals were removed by hydrochloric acid (HCl) and hydrofluoric acid (HF). 15 g coal (200 meshes) was wetted with 3.0 mL ethanol, then 80 mL of 5 mol/L HCL and 80 mL of 40% HF were added to wetted coal. The mixtures were heating in water bath of 60 ℃ for 3 h. When the mixtures were cooled down to ambient temperature (25 ℃), the sample was washed to pH = 7 with ultrapure water and then was dried at 60 ℃ for 12 h.

### 2.2 Graphitization

Pulverized coal was mixed with catalysts (4: 1 in weight), and then ground in by an agate mortar to below 200 meshes. The catalysts include H_3_BO_3_, Fe_2_(SO_4_)_3_, FeCl_3_, FeS_2_, and H_3_BO_3_+FeCl_3_, respectively. Then the mixture was placed into a graphite crucible with two pores on the lid. Then it was subjected to catalytic graphitization. Coal at ambient temperature was denoted as C-25, coal graphitized at 3000 ℃ was denoted as coal-based graphite (CBG), coal mixed with catalysts graphitized at 3000 ℃ were denoted as CBG + catalyst, where the catalysts were H_3_BO_3_, Fe_2_(SO_4_)_3_, FeCl_3_, FeS_2_, and H_3_BO_3_+FeCl_3_. Samples were heated to 3000 ℃ at 10 ℃/min and held for 3 h, naturally cooled to room temperature. The heating atmosphere was inert argon.

### 2.3 Experimental methods

#### 2.3.1 X-ray diffraction (XRD) analysis.

**Fig 1:**
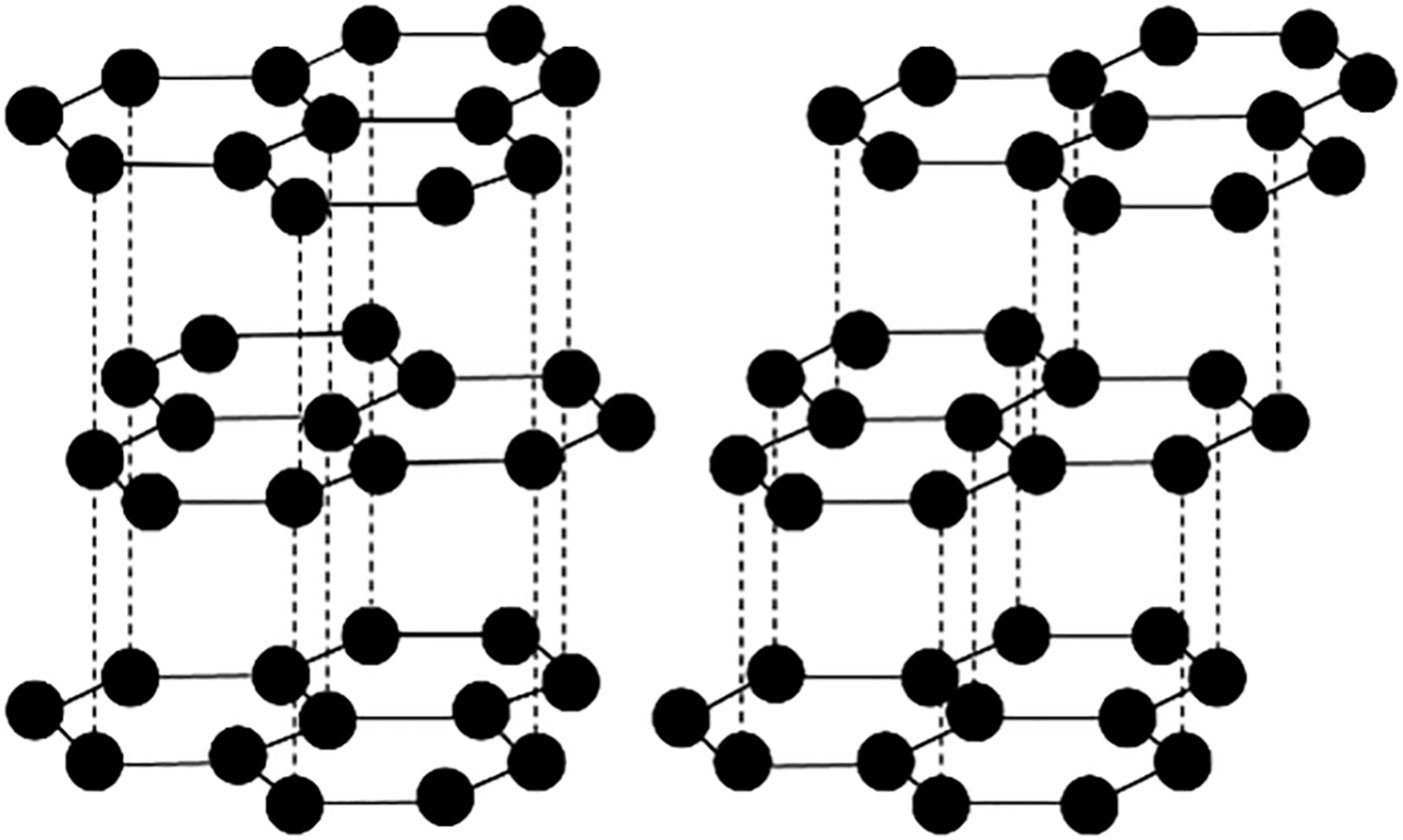
The arrangements of 2H (left) and 3R (right) structures of graphite.

**Fig 2:**
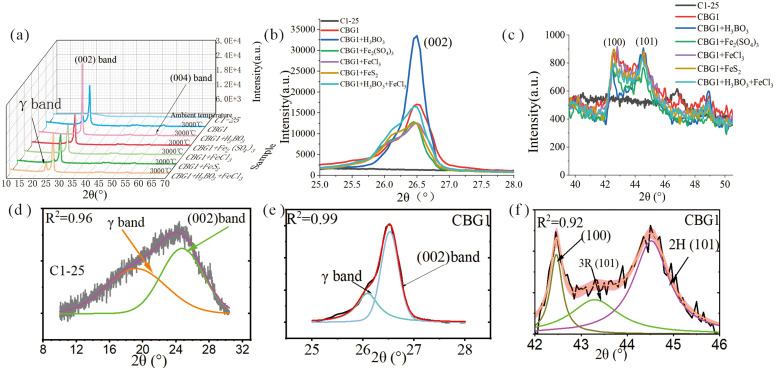
XRD spectra of samples and peak fitting curves. (a) XRD spectra of all graphitized samples; (b) the (002) band of all graphitized samples; (c) the (100) and (101) bands of all graphitized samples; (d) fitted peaks of (002) band of sample C1-25; (e) fitted peaks of (002) band of sample CBG1; (f) fitted peaks of (100) band and (101) band of sample CBG1.


The XRD was conducted using Bruker D8 Advance equipped with Cu Kα radiation (λ = 0.154056 nm). Samples were scanned continuously from 10 to 70 ° at a speed of 3 °/min, after calibrating the instrument by using a standard silicon powder. The sampling width was 0.02 °. The asymmetric bands in XRD patterns were curve-fitted using the Voigt function for graphitized sample and Gaussian function for coal at ambient temperature by Origin software, the iteration number was set until all peaks were converged. The average structural parameters of crystalline, the spacing between (hkl) planes (d_002_), crystallite size along c direction (L_c_) and along a direction (L_a_), aromaticity (f_ɑ_), graphitization degree (G), and layers of graphene (N) were determined and calculated by equations (1) to (6), respectively [1,21,22]. Where f_ɑ_ was also an index of proportion of amorphous carbon and graphitic carbon. Additionally, the area ratio (2H/3R) of ABA hexagonal phase/ABCA rhombohedral phase of graphite was calculated by curve-fitting process [4,20]. Because the (002) peak near 26.38 ° of the 2H phase was not easily resolved from the (003) peak nearby 26.60 ° of the 3R phase, the (101) peaks from 2H and 3R phases near 45 ° were distinguishable [4] and were chosen to present the 2H and 3R phase. Peaks were fitted in Origin software with R^2^ above 0.9. The arrangements of 2H and 3R structures of graphite are shown in Fig 1. The detailed procedures of fitted peaks were shown in Fig 2, taking coal 1 at ambient temperature and 3000 ℃ as an example.

$$d_{\text{0}\text{0}\text{2}}=\frac{\lambda }{\left(\text{2}\cdot \text{sin}\theta_{\text{0}\text{0}\text{2}}\right)}$$ (1)

$$L_{a}=\text{1}.\text{8}\text{4}\cdot \frac{\lambda }{\left(\beta_{\text{1}\text{1}\text{0}}\cdot \text{cos}\theta_{\text{1}\text{1}\text{0}}\right)}$$ (2)

$$L_{c}=\text{0}.\text{8}\text{9}\cdot \frac{\lambda }{\left(\beta_{\text{0}\text{0}\text{2}}\cdot \text{cos}\theta_{\text{0}\text{0}\text{2}}\right)}$$ (3)

$$f_{a}=\frac{A_{\text{0}\text{0}\text{2}}}{\left(A_{\text{0}\text{0}\text{2}}+A_{\gamma }\right)}$$ (4)

$$G=\text{1}\text{0}\text{0}\cdot \frac{\text{0}.\text{3}\text{4}\text{4}\text{0}-d_{\text{0}\text{0}\text{2}}}{\text{0}.\text{3}\text{4}\text{4}\text{0}-\text{0}.\text{3}\text{3}\text{5}\text{4}}$$ (5)

$$N=\left(\frac{Lc}{d_{\text{0}\text{0}\text{2}}}\right)+\text{1}$$ (6)

Where *λ* is the Cu Kα wavelength (0.154056 nm) of the used radiation; $\beta_{\text{1}\text{1}\text{0}}$ and $\beta_{\text{0}\text{0}\text{2}}$ are the full-width at half maximum (FWHM) of (110) and (002) peaks, $A_{\gamma }$ and $A_{\text{0}\text{0}\text{2}}$ are areas of the amorphous band and (002) peak.

#### 2.3.2 High resolution transmission electron microscopy (HR TEM) observation.

The HRTEM observations were performed on a JEM-F200 field emission transmission electron microscope, with an accelerating voltage of 200 kV. The line resolution was below 0.1 nm, and the point resolution was below 0.23 nm. Samples below 200 meshes were diluted with ethanol and dispersed for 15 min via ultrasonic oscillations. After ultrasonic oscillations, the suspended samples were transferred onto the copper grid for observation with suitable magnification.

#### 2.3.3 Electrical conductivity measurement.

Electrical conductivity (σ) of coal and graphitized samples were measured using the standard four-probe technique of FT-301b intelligent powder resistivity test system. The current accuracy and resistance were 0.1% and 0.3%, at room temperature. Two parallel measurements were conducted with error below 2.0%.

## 3 Results and discussion

### 3.1 Average crystalline structural analysis by XRD

XRD is a common method to investigate the average structural order of carbonaceous materials [20], in terms of visual XRD patterns [7,23–25] and quantitative structural parameters including d_002_, L_a_, L_c_, N, fα and G [22,26]. Coal at ambient temperature showed a broad and weak (002) band (Fig 1a), the asymmetric (002) band was curve-fitted into γ band and (002) peak (Fig 1d), indicating the existence of a great amount of amorphous and turbostratic structures [20,27]. When coal was graphitized, the (002) peak became sharp and narrow (Figs 1a, e). The (002) peak of graphitized coal mixed with boric acid showed stronger intensity than graphitized coal without catalyst and with catalyst including Fe_2_(SO_4_)_3_, FeCl_3_, FeS_2_, and H_3_BO_3_+FeCl_3_. The strong and narrow (002) peak indicates a high degree of stacking in the vertical of graphene plane [1], resulting in a high degree of crystallinity. Nevertheless, the weak asymmetry of (002) band still existed in all graphitized samples, indicating the existence of non-graphitic or multiple graphitic structures [27], as observed the coexistence of kinds of nanostructures [11] and micro petrological graphitic components [28]. The (002) band of graphitized coal and natural graphite composed of turbostratic and graphite structures [27], where the turbostratic structure resulted from lattice distortion and was also named disordered structure [7] and pseudocrystallite [26], with average interlayer space of 0.3440 nm to 0.3354 nm along c-axis. Comparing to (002) reflection, the intensities of (100), (101), (004) reflections were relatively weak, nevertheless, these reflections provided crucial information of aromatic structures [4,27]. Three peaks (Fig 1f), including 2H phase (100), 3R phase (101), and 2H phase (101) were fitted at the ranges of 2θ = 42 ° to 46 °. The 2H/3R was the area ratio of 2H phase (101) to 3R phase (101). The intensity and area of 2H phase (101) were stronger and larger than 3R phase (101), indicating a larger proportion of 2H phase graphite than 3R phase graphite, similarly to these commercial graphene-based materials [4]. The 2H phase graphite is usually more stable than the 3R phase graphite [27], because the 3R phase graphite is frequently transformed to the 2H phase graphite with metamorphism and heat treatment [4,29].

Coals at ambient temperatures have d_002_ values above 0.3440 nm [22,30]. As shown in Table 1, d_002_ values decreased from 0.3566 nm to 0.3449 nm with rising metamorphism (from sub-bituminous coal to anthracite). When coal was graphitized, the d_002_ values were below 0.3440 nm, where the 0.3440 nm was viewed as the threshold of graphitic carbon and non-graphitic carbon [1]. The minimum d_002_ of graphitized coal was 0.3355 nm, very close to d_002_ of the ideal graphite (0.3354 nm). The maximum graphitization degree (G) calculated from d_002_ was 98.64%. However, the d_002_ and G were average parameters of bulk sample, multiple carbon nanostructure and micro petrological components coexisted both in natural coaly graphite [22,28] and coal-based graphite [11], as observed in the asymmetric (002) band. Therefore, the aromaticity (f_ɑ_), calculated from curved-fitted γ band and (002) peak, was a crucial parameter to judge the proportion of graphite and non-graphite [4,6,20]. The f_ɑ_ values of graphitized coal mixed with boric acid were quite larger than graphitized coals without any catalysts and coals with catalysts of Fe_2_(SO_4_)_3_, FeCl_3_, FeS_2_, and H_3_BO_3_+FeCl_3_. From the nanostructure point of view, the proportion of graphite structure in boric acid catalyzed samples was more than graphitized raw coal and Fe_2_(SO_4_)_3_, FeCl_3_, FeS_2_, and H_3_BO_3_+FeCl_3_ catalyzed coals, as also revealed by larger Lc and N, higher content of graphite in Table 1. The boric acid catalyzed organic material owned high graphitization degree and presented ideal electrochemical performance [2], which was the potential anode materials.

**Table 1:** The calculated crystallite parameters of coal and graphitized samples.

Sample	d_002_(nm)	La(nm)	Lc(nm)	fɑ(%)	G(%)	2H/3R	N(layer)
C1-25	0.3566	2.17	1.37	46.11	–	–	5
CBG1	0.3364	24.83	18.20	71.48	88.29	3.42	55
CBG1 + H_3_BO_3_	0.3368	34.44	28.81	81.30	84.04	5.94	87
CBG1 + Fe_2_(SO_4_)_3_	0.3385	24.94	13.09	75.88	64.43	4.51	40
CBG1 + FeCl_3_	0.3381	17.37	12.62	79.31	68.14	1.46	38
CBG1 + FeS_2_	0.3384	28.78	12.30	78.52	65.05	4.09	37
CBG1 + H_3_BO_3_+FeCl_3_	0.3380	20.37	12.97	78.98	69.42	2.26	39
C2-25	0.3492	2.97	2.14	79.96	–	–	7
CBG2	0.3356	12.18	26.49	81.19	97.58	1.51	80
CBG2 + H_3_BO_3_	0.3356	14.10	30.98	80.37	97.88	2.19	93
CBG2 + Fe_2_(SO_4_)_3_	0.3362	21.96	19.35	82.04	90.80	2.91	59
CBG2 + FeCl_3_	0.3355	17.85	22.59	80.78	98.64	3.20	68
CBG2 + FeS_2_	0.3361	27.53	22.88	80.72	92.44	5.27	69
CBG2 + H_3_BO_3_+FeCl_3_	0.3356	34.24	26.00	81.47	98.08	4.42	78
C3-25	0.3449	2.14	2.24	67.67	–	–	7
CBG3	0.3381	24.44	18.24	79.62	69.11	5.41	55
CBG3 + H_3_BO_3_	0.3357	36.67	23.95	80.24	96.56	5.09	72
CBG3 + Fe_2_(SO_4_)_3_	0.3377	33.24	16.74	80.65	73.12	8.94	51
CBG3 + FeCl_3_	0.3379	44.03	19.64	80.20	70.68	8.70	59
CBG3 + FeS_2_	0.3375	35.44	18.02	81.13	75.88	6.14	54
CBG3 + H_3_BO_3_+FeCl_3_	0.3375	32.26	19.73	80.20	75.43	7.33	59

Note: C1-25, C2-25, C3-25 are coals at ambient temperature, CBG is coal-based graphite.

### 3.2 Morphology and nanostructure analysis by HR TEM

The aromatic lattice of coals at ambient temperature have been qualitatively described from the perspective of morphology and lattice fringes [31–34]. With increasing coal rank or metamorphism, the average lattice fringe length increases, and the alignment of lattice fringes becomes preferred, etc., consistent with those from XRD. Numerous studied [27,28,35–38] have addressed the characteristics of coals at ambient temperatures, which we will not elaborate on further here.

Four types of nanostructured carbon have been identified in coal-based graphite, namely amorphous, concentric, graphite-like, and pyrolytic nanostructures [11,28,34,39]. These four nanostructures correspond to non-graphitized carbon, difficultly-graphitized carbon, easily-graphitized carbon [11,40–42], and vapor-deposited carbon [43], respectively. Under HR TEM observation, the graphite-like carbons presented as multiple-layer lattice fringes, while the pyrolytic carbons were few-layer lattice fringes under HR TEM identification. The formation processes of these nanostructured carbon are explained by combination of HRTEM and XRD analyses [11]. However, amorphous nanostructures under HR TEM observation [11,39] are absent in present catalyzed graphitization, which might be attributed to catalyst-facilitated structural evolutions [1,2,16].

**Fig 3:**
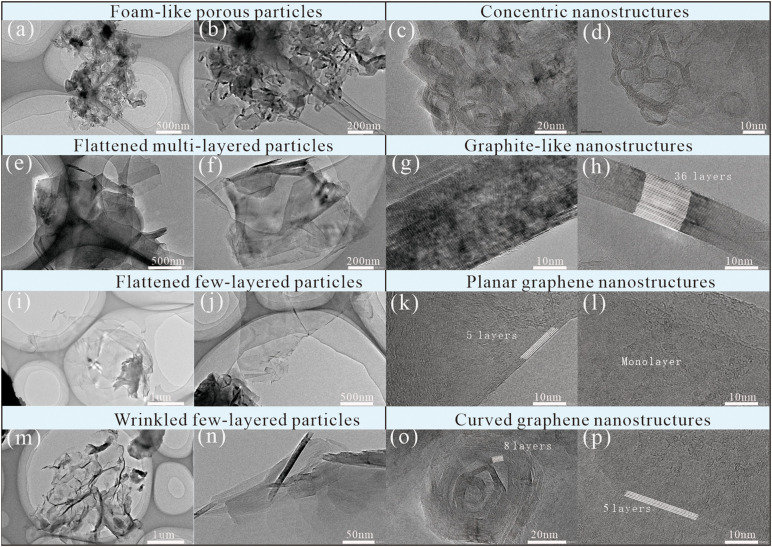
Morphologies of nanostructures in boric acid-catalyzed samples.

As shown in Fig 3, four distinct types of particles are identified in catalyzed graphitization via HR TEM characterization, namely foam-like porous particles, flattened multi-layered particles, flattened few-layered particles, and wrinkled few-layered particles. As shown in Fig 3, the foam-like porous particles (Fig 3A and 3B) were composed of concentric nanostructures (Figs 3C and 3D), characterized by highly porous framework and contributed to great capacity [7]. This type is also formed by successive graphitization from difficultly graphitizable carbon [11]. The flattened multi-layered particles (Figs 3E and 3F) correspond to graphite-like nanostructures (Figs 3G and 3H). HR TEM observations reveal dense stacking with well-ordered alignment, endowing high electronic conductivity. In contrast, flattened few-layered particles (Fig 3I and 3J) are dominated by planar graphene nanostructures (Fig 3K and 3L). HR TEM images clearly resolve 1–6 layers of graphene sheets with a planar, wrinkle-free morphology, and the interlayer spacing is slightly expanded due to the weak van der Waals forces between adjacent layers. This nanostructure retains the intrinsic electronic properties of graphene, including high carrier mobility, which is attributed to the absence of interlayer twisting and wrinkling-induced scattering [44,45]. Wrinkled few-layered particles (Fig 3M and 3N) are characterized by curved graphene (Fig 3O and 3P). HR TEM images reveal few layers of graphene sheets with obvious spontaneous curvature. This curved nanostructure is driven by the competition between stacking energy and deformation energy, offering opportunities for the application of coal-based materials in optoelectronic devices [46].

The structural differences between these particles and nanostructures exert a direct regulatory effect on properties. Specifically, concentric carbon nanostructures and curved graphene nanostructures stand out for enhancing high specific surface area and boosting mass transfer efficiency [7], whereas graphite-like nanostructures and planar graphene nanostructures play a dominant role in optimizing electronic conductivity [1]. This well-defined structure-performance correlation not only clarifies the intrinsic link between nanoscale morphology and functional behaviors but also provides a solid theoretical foundation for precisely tailoring the functional properties of target products through nanostructure modulation Fig 3.

### 3.3 Electrical conductivity and correlation on crystalline structure

**Fig 4:**
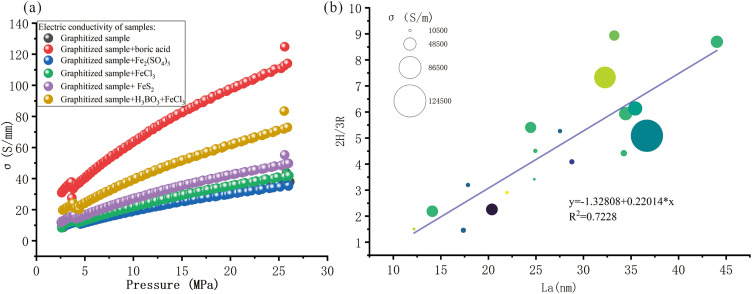
Electric conductivity of graphitized sample and the relationships with structural parameters. (a) electric conductivity of catalyzed graphitized samples; (b) the bubble plots of La, 2H/3R, and electric conductivity of catalyzed graphitized samples.

As shown in Fig 4, the electrical conductivity of boric acid catalyzed graphitized samples (red plots in Fig 4a) were three times than that of graphitized samples (yellow plots in Fig 4a), and also larger than the other catalyzed graphitized sample. For all samples, the electric conductivity exhibited a significant upward trend with increasing pressure, this phenomenon resulted from pressure-induced ordering of structures, facilitating charge carrier transport. The boric acid catalyzed graphitized samples showed the most prominent electrical conductivity enhancement. A comparison of this work with relevant literature is also presented in Table 2. A graphite powder with 7.7 MPa has lower electrical conductivity (only 0.290) than samples with the same pressure in this work, indicating the effective catalyzed graphitization.

**Table 2:** Comparison of electrical conductivity of this work and literatures.

Materials	Test condition	б(S/cm)	Test condition	б(S/cm)	Reference
CBG1	26MPa	10.505	7.7MPa	4.546	This work
CBG1 + H_3_BO_3_		51.427		21.529	
CBG1 + Fe_2_(SO_4_)_3_		16.823		6.703	
CBG1 + FeCl_3_		19.902		8.202	
CBG1 + FeS_2_		20.092		7.974	
CBG1 + H_3_BO_3_+FeCl_3_		45.220		18.566	
CBG2		11.405		6.032	
CBG2 + H_3_BO_3_		44.664		25.252	
CBG2 + Fe_2_(SO_4_)_3_		13.308		7.106	
CBG2 + FeCl_3_		16.654		10.879	
CBG2 + FeS_2_		16.952		8.964	
CBG2 + H_3_BO_3_+FeCl_3_		24.740		13.482	
CBG3		43.520		16.288	
CBG3 + H_3_BO_3_		124.809		53.780	
CBG3 + Fe_2_(SO_4_)_3_		40.039		15.909	
CBG3 + FeCl_3_		47.645		18.423	
CBG3 + FeS_2_		55.288		22.845	
CBG3 + H_3_BO_3_+FeCl_3_		83.437		32.969	
natural graphite1	1.5 Kg	1.384	–	–	[47]
natural graphite2		4.124	–	–	
natural graphite3		4.43	–	–	
natural graphite4		5.668	–	–	
natural graphite5		8.702	–	–	
graphite powder	–	–	7.7MPa	0.290	[48]

The electrical conductivity of carbonaceous materials was related to crystallinity, lateral dimension, and even the spacing of layers, whereas non direct relationships were visualized. Instead, here the bubble plots of electrical conductivity, 2H/3R proportion, and La, were plotted in Fig 4b. A linear correlation was observed between La and 2H/3R ratio, with the fitting equation y = −1.32802 + 0.22014x, and a coefficient of determination R^2^ = 0.7228. This indicated a non-negligible intrinsic linkage between the in-plane crystallite size and 2H/3R polytypic graphite. The coexistence of 2H/3R polytypic graphite synergistically enhanced the electrical conductivity behavior of graphitized samples. The ordered 2H phase provided a continuous charge transport pathway, while the 3R phase modulated interlayer stacking to reduce carrier scatting.

## Conclusion

Coal graphitization with catalysts of boric acid, Fe_2_(SO_4_)_3_, FeCl_3_, FeS_2_, and H_3_BO_3_+FeCl_3_, was studied. XRD and HR TEM were conducted on graphitized coal samples for crystalline structural analyses. The crystalline structure and electrical conductivity were also discussed, with a focus on correlating microstructure to conductivity and comparing the catalytic performance of different catalysts. The main conclusions are as follows.

Boric acid is a friendly catalyst for coal graphitization. Boric acid catalyzed samples present larger crystallite size (La, Lc) and higher proportion of 2H phase graphite than non-catalyzed samples. The chemical structure-electrical performance correlation analysis is confirmed and verified the practical value of boric acid catalyzed coal graphitization. A linear relationship between La and 2H/3R is established, confirming the intrinsic correlation of graphite-like nanostructure and average crystallite size.

In catalyzed samples, four types of particles associated with distinct nanostructures are observed: foam-like porous particles (correlated with concentric nanostructures), flattened multi-layered particles (correlated with graphite-like nanostructures), flattened few-layered particles (correlated with planar graphene nanostructures), and wrinkled few-layered particles (characterized by curved graphene nanostructures). Graphite-like nanostructures and planar graphene nanostructures are the key contributors to electrical conductivity.

## Supporting information

S1 File All data are included in the manuscript.
(DOCX)
