# Supplementary material for: Enhancing electrical conductivity of coal-derived graphite by boric acid catalysis: Correlating 2H/3R phase ratio with crystallite structure
Source: PLoS One. 2026 Apr 24;21(4):e0347483. doi: 10.1371/journal.pone.0347483 (PMC13108771; doi:10.1371/journal.pone.0347483)

Original and curve-fitted XRD spectra of coal at ambient temperature, taking coal 1 (C1-25) as an example


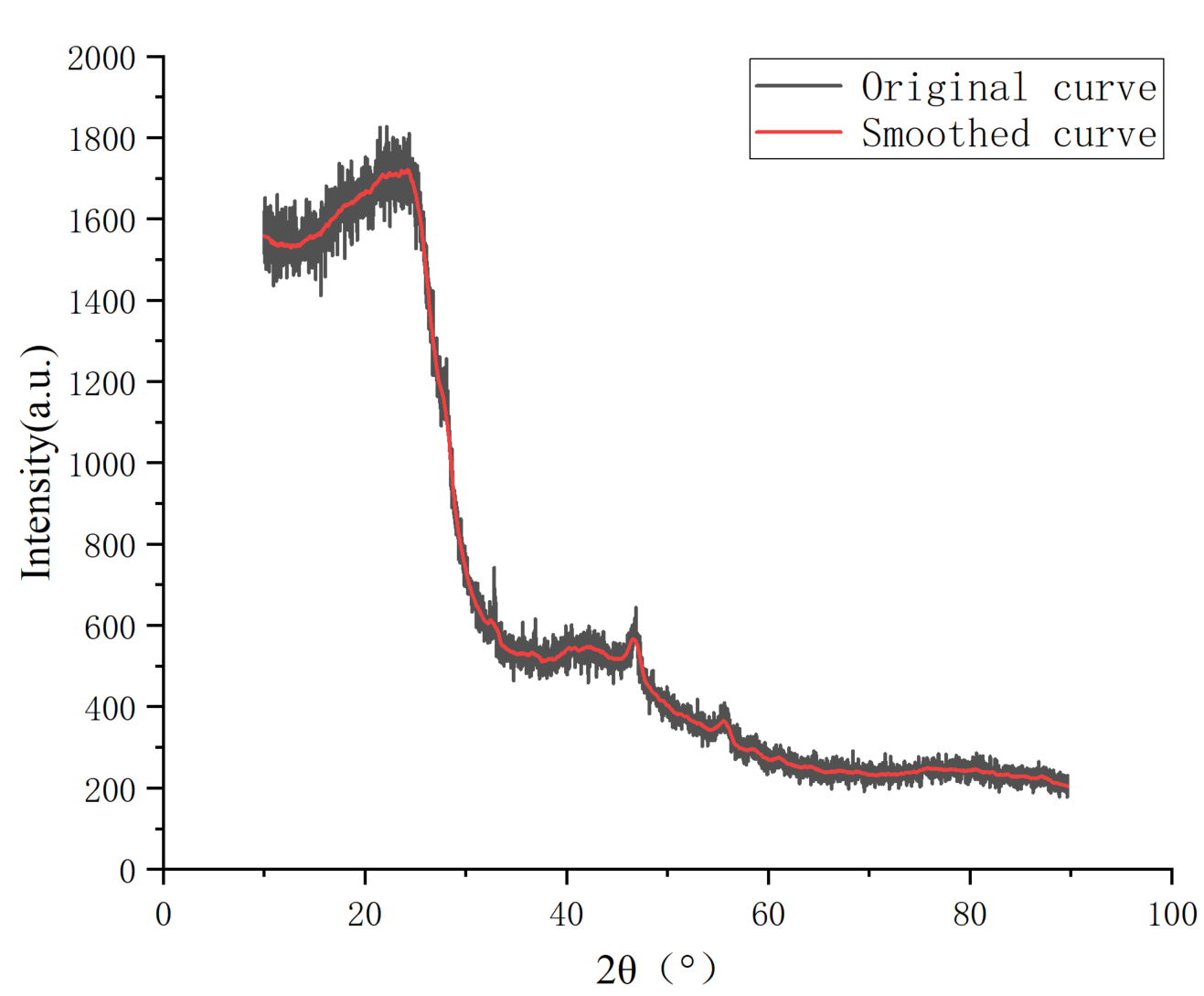

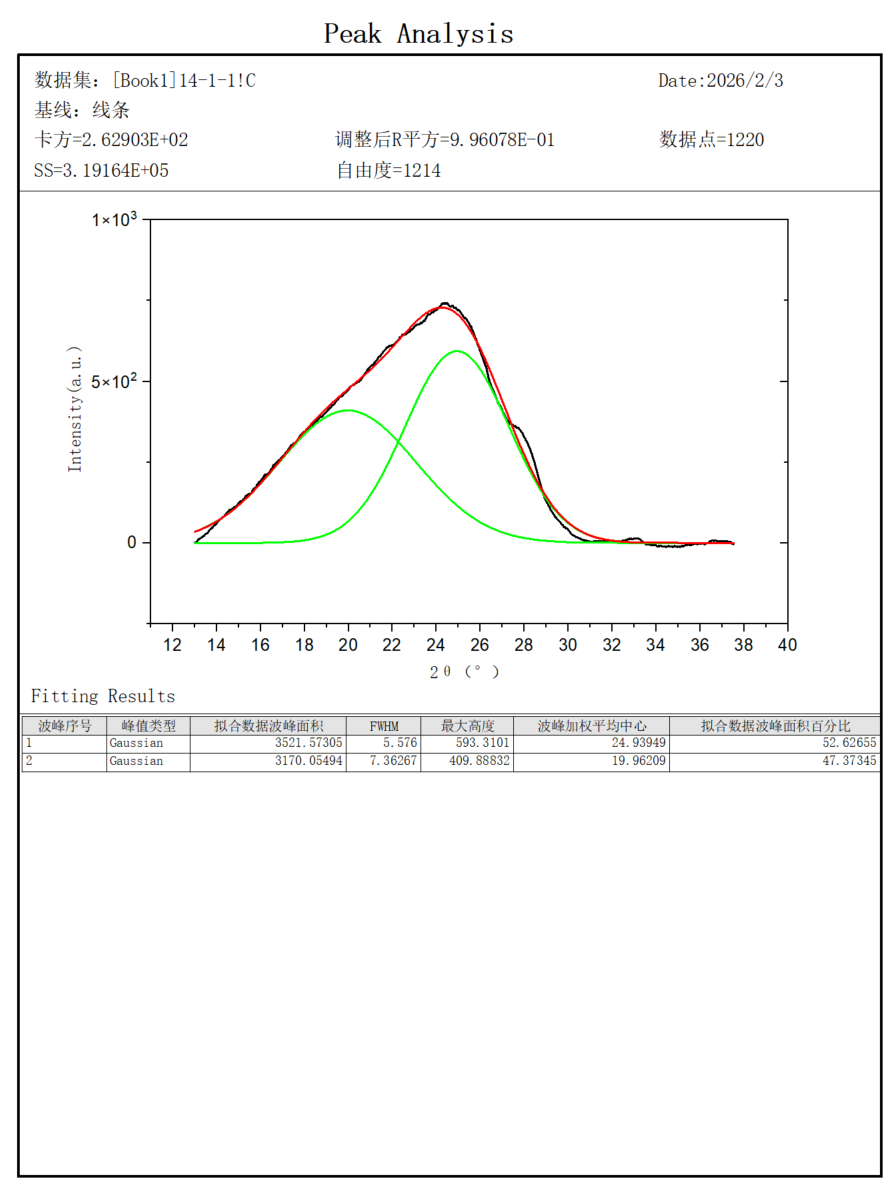

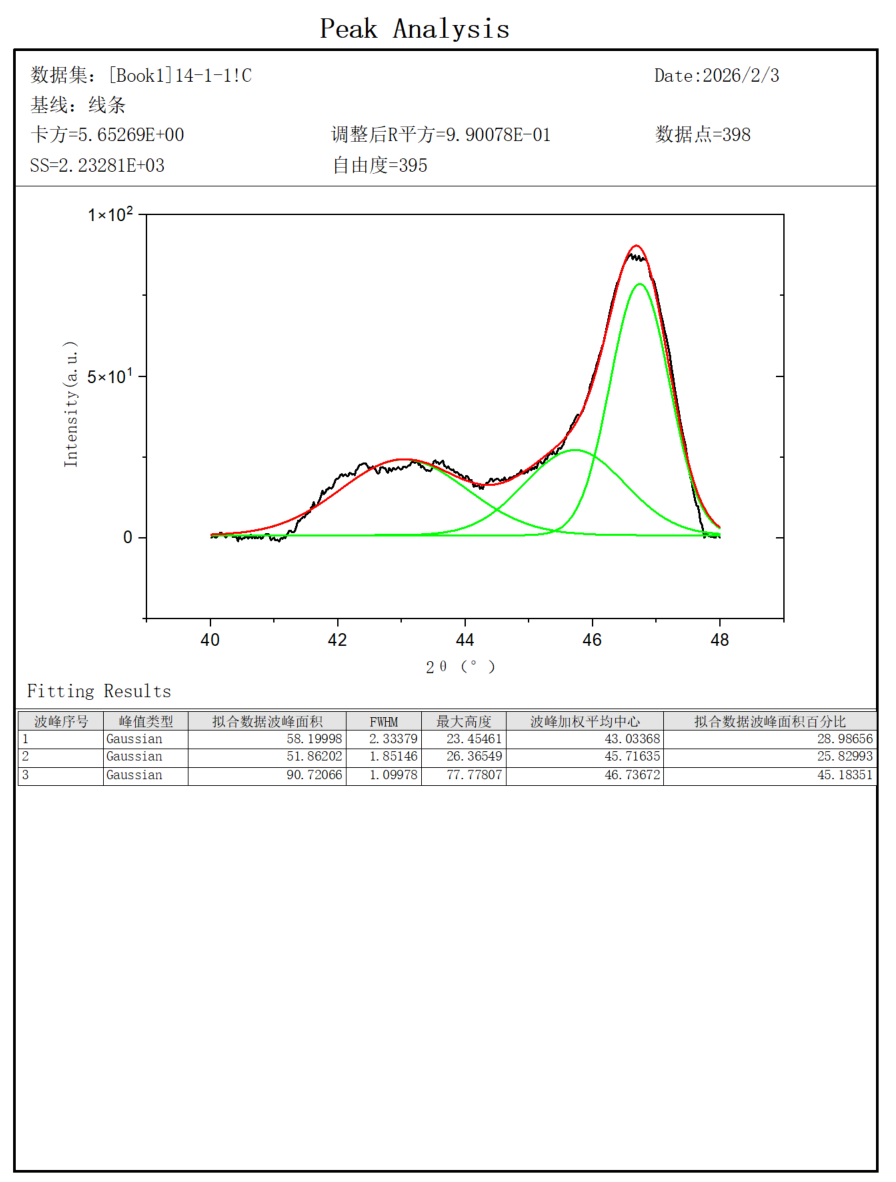


Original and curve-fitted XRD spectra of graphitized sample at 3000 ℃ (CBG1)


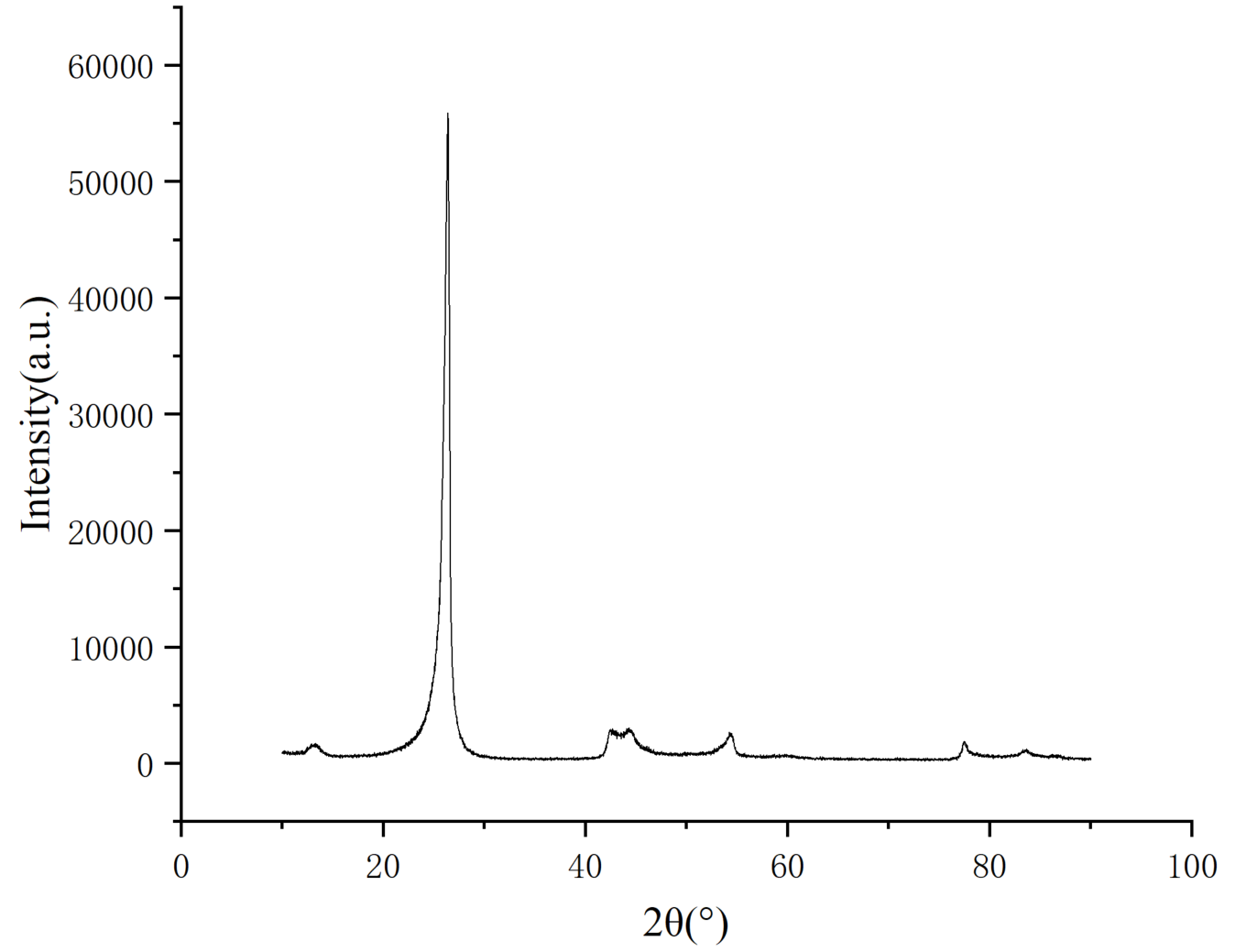

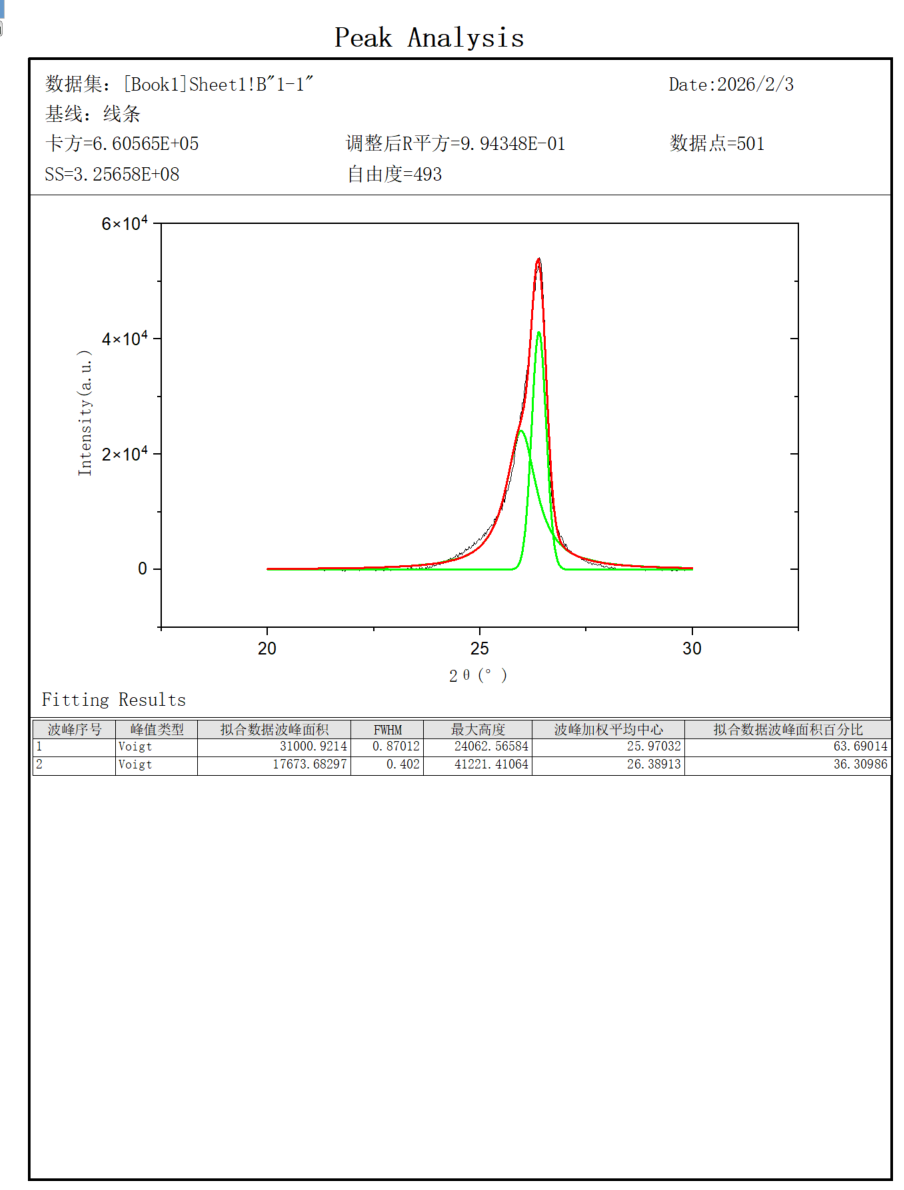

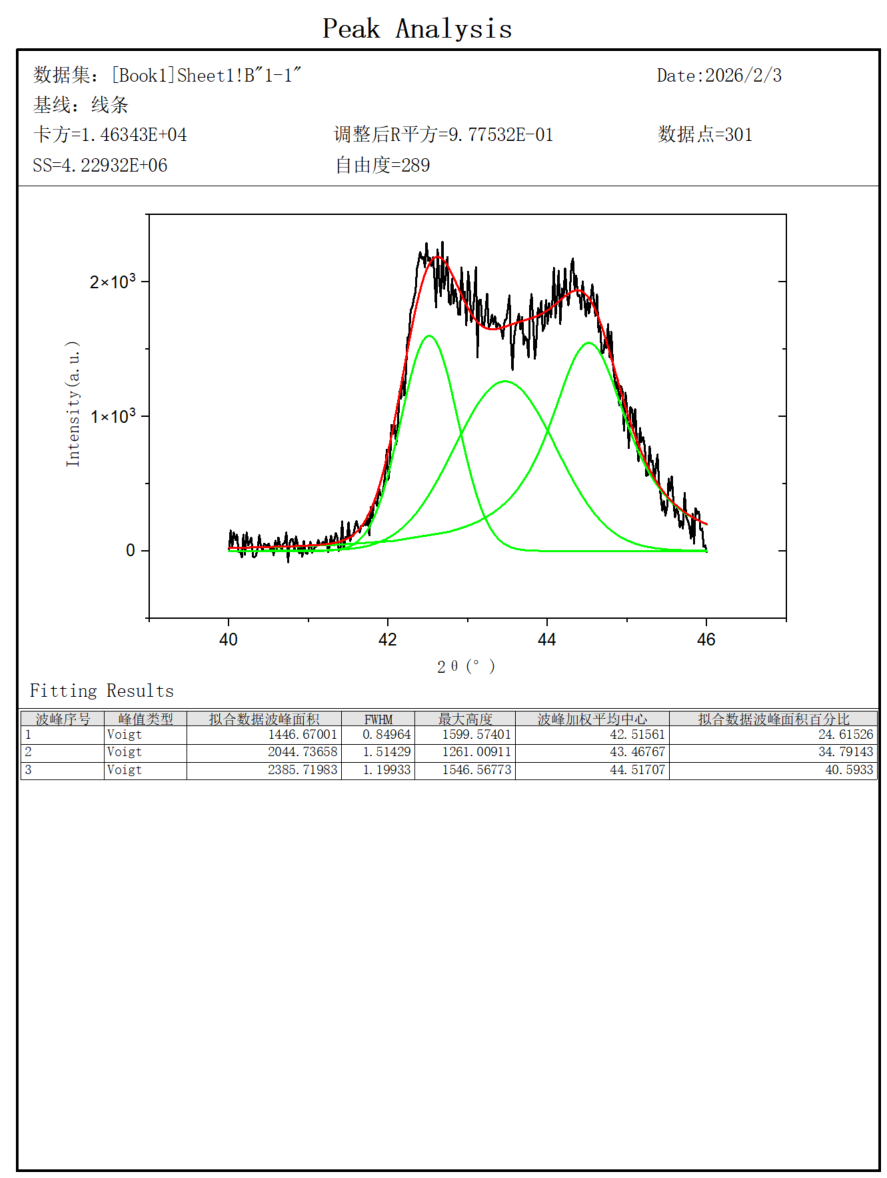


Reports of fitting residuals and goodness-of-fit parameters (R^2^＞0.9) of CBG1


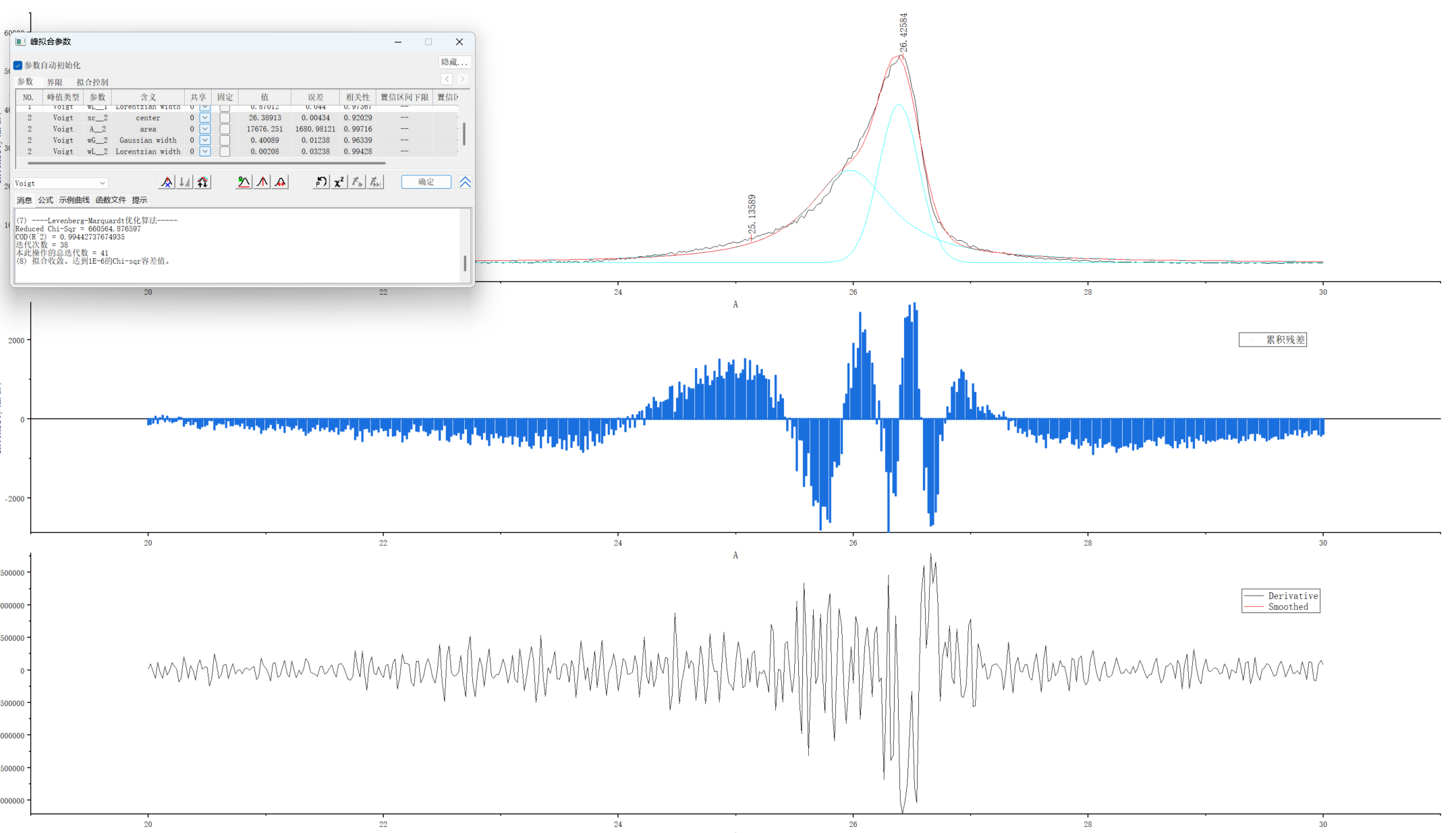

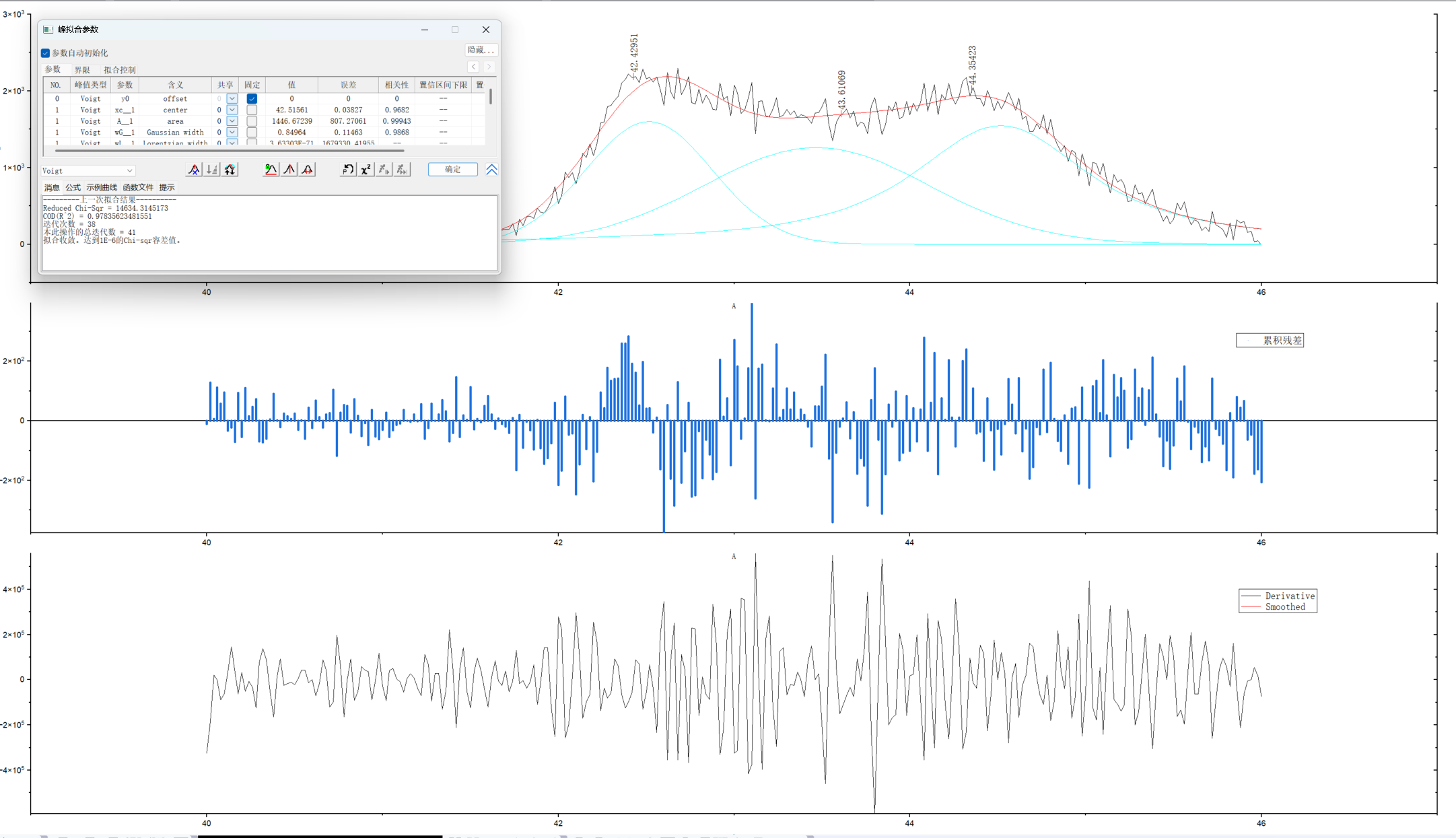

Supplement: S1 File — All data are included in the manuscript. (DOCX) [file pone.0347483.s001.docx]
